# Supplementary figures and images for: Muscarinic receptor M3 contributes to intestinal stem cell maintenance via EphB/ephrin-B signaling
Source: Life Sci Alliance. 2021 Jul 9;4(9):e202000962. doi: 10.26508/lsa.202000962 (PMC8321669; doi:10.26508/lsa.202000962)

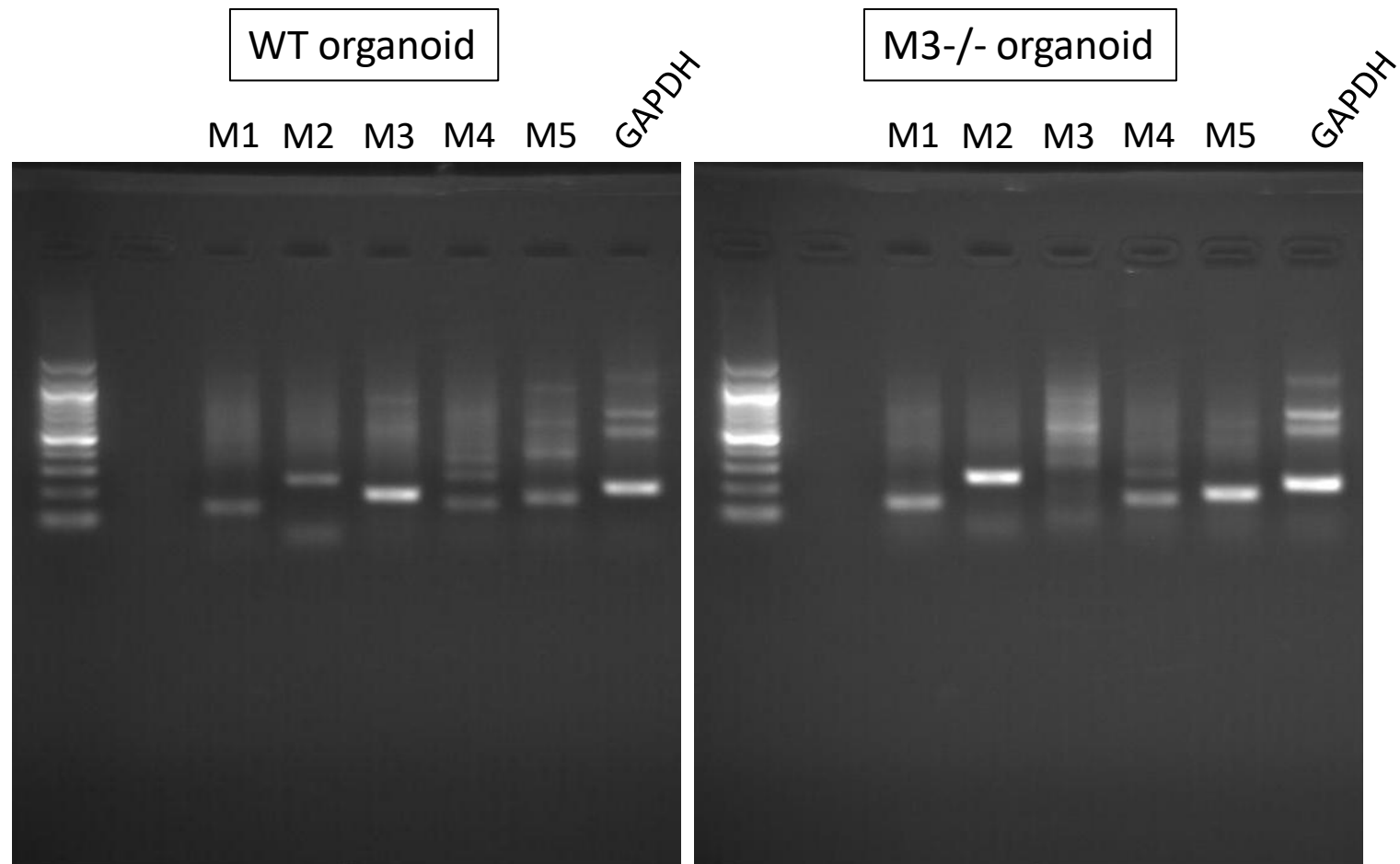

Fig 3A

Supplement: Supplementary file 4 [file LSA-2020-00962_SdataF3.zip › Fig 3 (Sourse Data)/Fig 3A.pdf]
